# Identification of atypical sleep microarchitecture biomarkers in children with autism spectrum disorder

**DOI:** 10.3389/fpsyt.2023.1115374

**Published:** 2023-04-17

**Authors:** Caroline Martinez, Zhe Sage Chen

**Affiliations:** ^1^Department of Pediatrics, Division of Developmental Pediatrics, Icahn School of Medicine at Mount Sinai, Kravis Children’s Hospital, New York, NY, United States; ^2^Department of Psychiatry, Department of Neuroscience and Physiology, Neuroscience Institute, New York University Grossman School of Medicine, New York, NY, United States

**Keywords:** sleep, machine learning, autism, spindle coupling, aperiodic component, REM sleep

## Abstract

**Importance:**

Sleep disorders are one of the most frequent comorbidities in children with autism spectrum disorder (ASD). However, the link between neurodevelopmental effects in ASD children with their underlying sleep microarchitecture is not well understood. An improved understanding of etiology of sleep difficulties and identification of sleep-associated biomarkers for children with ASD can improve the accuracy of clinical diagnosis.

**Objectives:**

To investigate whether machine learning models can identify biomarkers for children with ASD based on sleep EEG recordings.

**Design, setting, and participants:**

Sleep polysomnogram data were obtained from the Nationwide Children’ Health (NCH) Sleep DataBank. Children (ages: 8–16 yrs) with 149 autism and 197 age-matched controls without neurodevelopmental diagnosis were selected for analysis. An additional independent age-matched control group (*n* = 79) selected from the Childhood Adenotonsillectomy Trial (CHAT) was also used to validate the models. Furthermore, an independent smaller NCH cohort of younger infants and toddlers (age: 0.5–3 yr.; 38 autism and 75 controls) was used for additional validation.

**Main outcomes and measures:**

We computed periodic and non-periodic characteristics from sleep EEG recordings: sleep stages, spectral power, sleep spindle characteristics, and aperiodic signals. Machine learning models including the Logistic Regression (LR) classifier, Support Vector Machine (SVM), and Random Forest (RF) model were trained using these features. We determined the autism class based on the prediction score of the classifier. The area under the receiver operating characteristics curve (AUC), accuracy, sensitivity, and specificity were used to evaluate the model performance.

**Results:**

In the NCH study, RF outperformed two other models with a 10-fold cross-validated median AUC of 0.95 (interquartile range [IQR], [0.93, 0.98]). The LR and SVM models performed comparably across multiple metrics, with median AUC 0.80 [0.78, 0.85] and 0.83 [0.79, 0.87], respectively. In the CHAT study, three tested models have comparable AUC results: LR: 0.83 [0.76, 0.92], SVM: 0.87 [0.75, 1.00], and RF: 0.85 [0.75, 1.00]. Sleep spindle density, amplitude, spindle-slow oscillation (SSO) coupling, aperiodic signal’s spectral slope and intercept, as well as the percentage of REM sleep were found to be key discriminative features in the predictive models.

**Conclusion and relevance:**

Our results suggest that integration of EEG feature engineering and machine learning can identify sleep-based biomarkers for ASD children and produce good generalization in independent validation datasets. Microstructural EEG alterations may help reveal underlying pathophysiological mechanisms of autism that alter sleep quality and behaviors. Machine learning analysis may reveal new insight into the etiology and treatment of sleep difficulties in autism.

## Highlights

- *Questions*: Can a generalizable machine learning methodology identify sleep microarchitecture biomarker for children with autism spectrum disorder (ASD)?
- *Findings*: Using two public datasets (NCH dataset consisting of 149 ASD children and 197 age-matched controls (median age: 11 yr) without neurodevelopmental diagnosis; CHAT dataset consisting of 79 age-matched controls only), we extracted sleep macro- and microarchitecture features based on sleep EEG recordings and trained multiple machine learning models to predict the ASD risk for each patient. Our best model achieved a median area under the curve (AUC) of 0.93–0.98 in ten-fold, stratified cross-validated held-out data classification from the NCH dataset, and IQRAUC of 0.75–1.00 from the CHAT dataset. As an independent validation, our model showed good generalizability in a smaller NCH cohort of younger children population (median age: 2 yr., 38 autism and 75 controls).
- *Meaning*: Our results support that machine learning-driven model may discriminate ASD from healthy children based on identified sleep microarchitecture biomarkers. Future refinement of our model may help individualized diagnosis of ASD in pediatric practice.

## Introduction

Autism spectrum disorder (ASD) is a neurodevelopmental disorder characterized by social communicative deficits, restricted and repetitive behaviors and interests, and sensory sensitivities. Sleep problems or disorders are common, reportedly as high as 80% in ASD children, which can have a negative impact on children’s mental and physical health (1). Causes of sleep dysfunction in autism are thought to be related to biological, medical, behavioral dysfunction and are characterized by various sleep disturbances at night (1–3).

Electroencephalography (EEG) has been widely adopted in sleep analysis for ASD (4–6). Research has detailed reliable sleep macroarchitecture differences in ASD children: less total sleep time, decreased sleep efficiency, and decreased rapid-eye-movement (REM) sleep percent (7). Despite the high prevalence of sleep dysfunction and associated cognitive deficits in ASD children, only a few studies have examined the microarchitecture of sleep (8). Sleep microarchitecture consists of important periodic and aperiodic activities. Two important oscillatory activities include sleep spindles and slow waves during non-REM (NREM) sleep. Reduced spindle density has been shown in young children with ASD compared to age-matched children with developmental delay but without autism. (9) While sleep stages are associated with underlying predominant frequency oscillations. Sleep EEG signals also contain aperiodic features characterized by the 1/f component of power spectrum. Aperiodic signals have been linked to cognitive processes across all arousal states (10). Together, periodic and aperiodic spectral features exhibit sleep state dependent variations, which may reveal functional significance with changes in maturation and neurodevelopmental disorders (11).

Machine learning has become increasingly popular in clinical diagnosis and biomarker discovery including for children with neurodevelopmental disorders (12, 13). In this study, we hypothesized that the combination of machine learning and sleep EEG feature engineering can predict clinically diagnosed autism in children. We identified important sleep microarchitecture biomarkers that distinguish children with ASD and children without neurodevelopmental diagnosis, and verified our approach using two public datasets based on cross-validation and an additional independent dataset. The goal of this study was twofold: to assess the utility of overnight EEG as a potential autism diagnostic tool, to better understand the sleep changes related to autism beyond parent report and measured macroarchitecture changes.

## Methods

### Study population and cohort

Sleep polysomnogram and demographic data were obtained from the Nationwide Children’s Health (NCH) Sleep DataBank. The NCH Sleep DataBank is housed within the National Sleep Research Resource (NSRR), which consists of 3,984 polysomnography studies and over 5.6 million clinical observations on 3,673 unique patients in 2018–2019. Sleep studies were acquired under standard care at NCH. The published polysomnogram files (PSG) contain the patient’s physiological signals as well as the technician’s assessment of the sleep stages and descriptions of additional irregularities The accompanying records of clinical data were extracted from the electronic health record., and are separated into encounters, medications, measurements, diagnoses, and procedures. The dataset was then deposited in the National Sleep Research Resource (NSRR) (14).

A total of 149 children with autism (8–16 years old) and 197 age-matched controls without neurodevelopmental diagnosis were selected in our analysis. Sleep micro-and macroarchitecture undergo maturational changes across the lifespan with marked differences in infancy and late adolescence as sleep becomes more adultlike. For this reason, we chose the period of middle childhood to early adolescence.

Our selection criterion was based on ICD 10 codes present in clinical history. ICD 10 codes F84.0 were used to identify autism cases. Clinical diagnosis of autism are coded by physicians in the medical record by using the ICD code F84.0. This code encompasses DSMV criteria for autism including persistent impairment in reciprocal social communication and social interaction, and restricted, repetitive patterns of behavior, interests, or activities.

General exclusion criteria were neurological conditions including seizure disorder. The matched controls had no evidence of neuropsychiatric or neurodevelopmental disorders in ICD codes. ICD codes used for exclusion include ADHD, Intellectual Disability, Epilepsy, Cerebral Palsy, Global Developmental Delay, Language Delay, Motor Delay, and Genetic Syndromes.

Demographic variables (e.g., age at sleep study, gestational age, and gender) were further extracted. For independent dataset validation, we selected 79 age-matched patients from the Childhood Adenotonsillectomy Trial (CHAT) study, which was initially derived from a multi-center treatment trial for children with obstructive sleep apnea (15). This trial was a single-blind, randomized controlled trial that recruited children with symptoms of Obstructive Sleep Apnea from primary care, otolaryngology, and sleep medicine clinics at 7 academic sleep centers (Children’s Hospital of Philadelphia, Philadelphia, PA; Cincinnati Children’s Medical Center, Cincinnati, OH; Kosair Children’s Hospital (KCH), Louisville, KY; Rainbow Babies and Children’s Hospital, Cleveland, OH; Children’s Hospital, Boston, MA; Cardinal Glennon Children’s Hospital, St. Louis, MO; and Montefiore Medical Center, Bronx, NY). Children had baseline and 7 month follow-up polysomnographic, cognitive, and behavioral testing and accompanying clinical and laboratory evaluations. In the study, patients were randomly assigned to early adenotonsillectomy or a strategy of watchful waiting. Polysomnographic, cognitive, behavioral, and health outcomes were assessed at baseline and at 7 months post treatment. We included participants’ data from the baseline assessment only so that it contained all patients who were pre-treated.

However, the CHAT dataset contains no autism patients. Furthermore, a smaller independent NCH dataset using younger patients (age: 0.5–3 years) was created for validation. The data collection was approved by application to NSRR. All human subjects gave consent for use of their data. Features for machine learning were extracted from dataset csv and edf files. Macroarchitecture features were extracted from sleep specialist annotations. The software code that was used to extract microarchitecture features from edf files is based on pre-trained classifiers https://github.com/raphaelvallat/yasa_classifier (16, 17).

A flowchart of our study is shown in Supplementary eFigure S1 Demographic statistics of all analyzed data are summarized in Table 1.

**Table 1:** Summary of demographic data from the selected study groups.

Characteristics	NCH autism	NCH controls	CHAT controls	Statistics NCH vs. NCH	Statistics NCH vs. CHAT
Number of subjects	149	197	79	n/a	n/a
Age—yr., median [IQR]	11.7 [7.9,14.7]	10.4 [7.2,12.8]	9.2 [8.0,9.9]	*t*-value = 2.3, *p* = 0.43(*t*-test)	*t* = 5.7, *p* = 0.73
Male gender (%)	82%	60%	60%	Chi = 11.7, *p* = 0.006 (chi-squared test)	Chi = 3.6, *p* = 0.05
Gestational age	32 [31,36]	35 [34,39]	unknown	*t*-value = 5.8, *p* = 1.0 (*t*-test)	n/a
Apnea–hypopnea index (AHI)	5.8 [0,8]	6.6 [0,6]	5.9 [0,9]	*t*-value = 1.1, *p* = 0.3 (*t*-test)	*t*-value =0.09 *p* = 0.9
Ethnicity					
African-American	19%		23%		51%
White	71%		61%		36%
Other	10%		16%		13%
Characteristics	Autism infant/toddler		NCH infant/toddler		Statistics
Number of subjects	38		75		n/a
Age—yr., median [IQR]	2.1 [1.9,2.5]		2.0 [1.5,2.5]		*t*-value = 1.4, *p* = 0.18 (*t*-test)
Male gender (%)	74%		63%		chi = 1.9, *p* = 0.16 (chi-squared *t*-test)
Gestational age	36.5 [36,39]		36.6 [36,39]	*t*-value = 0.16, *p* = 0.87 (*t*-test)	
Apnea–hypopnea index (AHI)	3.6 [0,4]		6 [0,7]		*t*-value = 1.5, p = 0.16 (*t*-test)
Ethnicity					
African-American	21%			22%	
White	58%			65%	
Other	21%			13%	

### Sleep macroarchitecture

We used the polysomnogram files to extract the duration of sleep stages. Polysomnographic recordings were scored by technologists according to the American Academy of Sleep Medicine (AASM) criteria (18). The annotation files from the NCH and CHAT databases included manually graded sleep stages in 30-s epochs. All studies contain overnight recordings divided into epochs with a 30 s window size. All experiments employed AASM staging, where NREM3 and NREM4 were merged into the N3 stage. The following sleep stage features were collected: total sleep time, sleep efficiency, percentage of time in each sleep stage (N1, N2, N3, and R), number of REM sleep episodes, arousal index, and apnea–hypopnea index. The mean sleep recording duration was 10.3 h. The EEG electrode labels were consistent with the International 10/20 system, and a total of 7 EEG recording channels were measured during sleep studies.

### Sleep microarchitecture

#### Spectral analysis

We processed the EDF files with the MNE-Python toolbox^1^. Raw EEG data was first downsampled to 100 Hz, followed by bandpass filtering between 0.1 and 40 Hz. We selected seven EEG channels (two frontal F3, F4, two central C3, C4, two occipital O1, O2, and one midline central CZ, and used M1 and M2 as respective reference electrodes: F3-M2, F4-M1, C3-M2, C4-M1, O1-M2, O2-M1, and CZ-O1) to examine spectral features from bilateral frontal, central, and occipital regions. We applied YASA, a Python-based toolbox^2^ to compute sleep-associated EEG features (19). We first computed the power spectral density, and then derive the relative power (defined as the ratio between the band-specific power and total power) in delta (1–4 Hz), theta (4–8 Hz), alpha (8–12 Hz), sigma (12–15 Hz), beta (15–30 Hz), and gamma (30–90 Hz) frequency bands. Power Spectral Densities (PSD) were estimated by Welch’s estimator that computed Fast Fourier Transform for above frequency bands. Each window size was one epoch containing one second of data. A 50% overlapping Kaiser window was applied to smooth the PSD data over 3 windows.

### Spindle characteristics

We used YASA to further detect slow (9–12 Hz) and fast (12–15 Hz) spindles. In order to meet the spindle criterion, all three parameters must satisfy threshold criteria within the same time window: (i) relative sigma power ≥ 0.2, (ii) root mean square ≥ RMS_mean + 1.5 *RMS_std, and (iii) moving correlation≥0.65. Spindles with a duration shorter than 0.5 s or longer than 2 s were discarded. Spindles with a duration shorter than 0.5 s or longer than 2 s were discarded. Spindles detected within 500 ms of one another on the same channel were considered a single spindle.

Spindle detection criteria were derived from default threshold values. (19) Next, sleep spindle densities (# per min) as well as discrete peak-to-peak amplitudes, mean frequencies, and durations for each subject and channel were computed. Slow oscillations (SO) are characterized by high amplitude oscillations between 0.1 and 1.25 Hz. To characterize spindle-slow oscillation (SSO) coupling, we used YASA to detect slow waves, and then calculated the phase-amplitude coupling across NREM2 and NREM3 based on epochs centered around the negative peak of the slow waves. A Hilbert transform was applied to extract the instantaneous phase angle of SO during maximal spindle amplitude. The mean circular phase and mean vector length (MVL) across NREM sleep were determined using the Pingouin software package^3^ (20). The MVL was scaled between 0 and 1, with 1 indicating that the sleep spindle is occurring at the preferential phase of the SO (i.e., its trough), and 0 indicating that the spindle is out of phase with SO. Additionally, we detected the phase of SO where the highest peak of spindle amplitude occurred. The SO trough was considered to be the trigger, with the spindle occurring within ±1.2 s.

### Aperiodic signals

The EEG power typically decreases with increasing frequencies, following a 1/f-like distribution. The aperiodic or 1/f component of the power spectrum can be quantified in terms of an intercept and a slope–the exponential decrease of power as a function of frequency (20, 21). The Fitting Oscillations and One Over f (FOOOF) toolbox^4^ was used to assess the spectral slope and the intercept (22).

### Machine learning analyses

We used Scikit-Learn’s Python library for the classification analysis (23). Three commonly used machine learning models: logistic regression (LR), support vector machine (SVM), and random forest (RF) were tested. In the LR model, we imposed LASSO (Least Absolute Shrinkage and Selection Operator) regularization. The hyperparameters in all classifiers were selected based on cross-validation among training samples. In model assessment, we used 10-fold stratified cross-validation with 20 repeats and reported the median performance statistics. The predictive performance was assessed in terms of accuracy, sensitivity, specificity, and F1 score. Because of sample imbalance between positive and negative classes, we used the area under the receiver operating characteristic curve (AUC) to determine the overall performance.

### Feature ranking

We grouped sleep EEG features into four categories: *spectral power, aperiodic features, microarchitecture,* and *sleep spindle characteristics*. Feature ranking was applied to reveal the relative importance of each feature. For the linear SVM classifier, the importance of each feature was determined by the associated weight. In LR, features were eliminated by shrinking the values of the coefficients of redundant features to 0. RF feature selection was implemented based on higher splits containing larger information gains. Furthermore, each classification analysis was rerun by discarding one set of features (i.e., leave-one-feature-set-out) to assess the relative performance loss.

### Independent data validation

In addition to cross-validation in the NCH dataset, we further validated confirmed our method using an independent pediatric sleep study (CHAT). As the CHAT study contains no autism data, ASD patients from the NCH dataset was used to match with CHAT controls. A second validation set was also constructed from the NCH study using younger children with autism and normally developing children. Machine learning models were generated using these separate datasets.

## Results

### Study population

Table 1 compares demographic sample characteristics of three studied datasets. The age of patients from NCH autism, NCH control, and CHAT control groups was not significantly different between each other. However, there was a higher percentage of males in the NCH autism group. This was not very surprising given a ratio of 4:1 male-to-female predominance in autism26. Gestational age was comparable between NCH autism patients and controls, but not available for CHAT controls. Obstructive hypopnea was not significantly different between groups.

### Feature ranking

**Figure 1:**
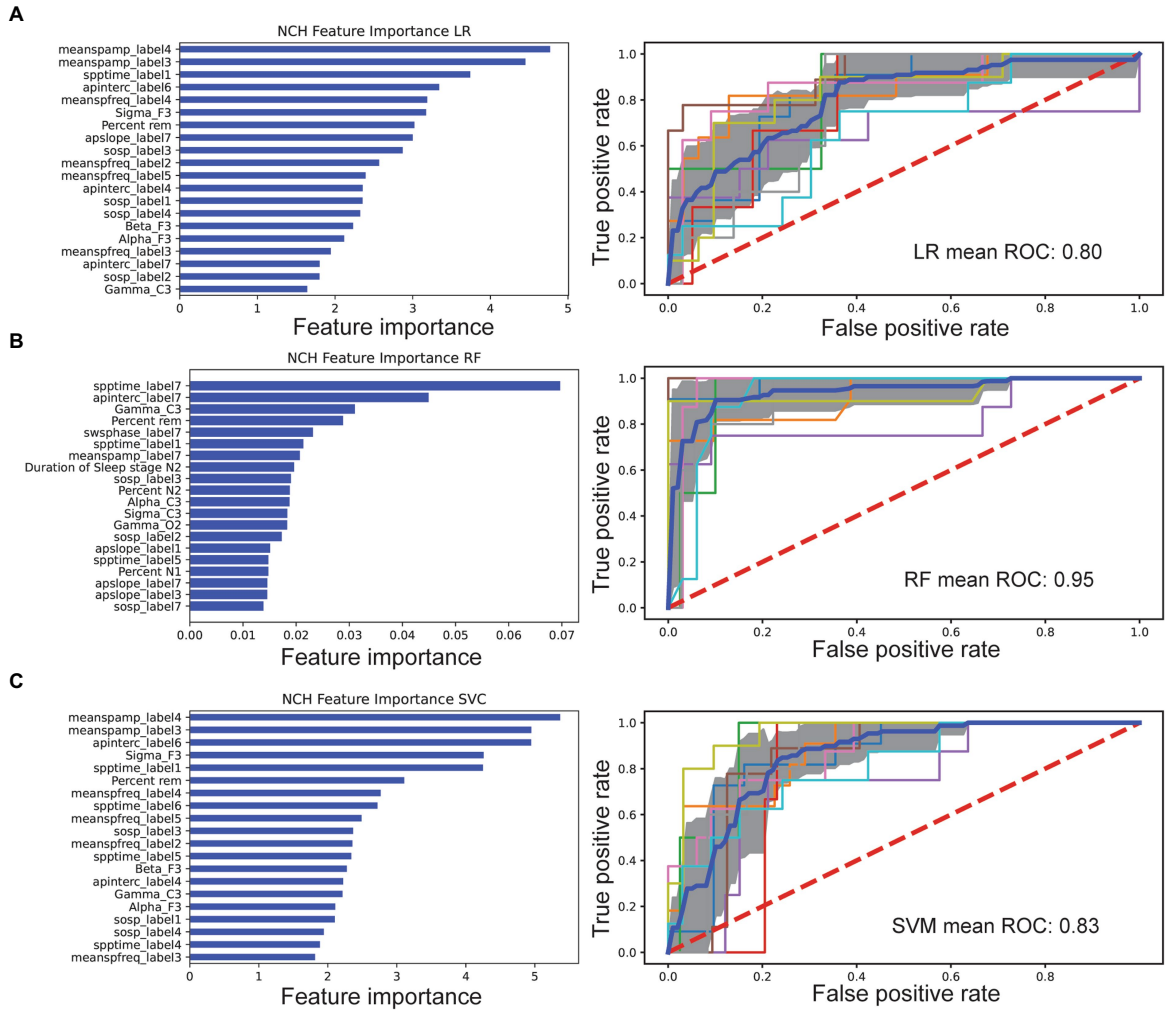
Comparison of feature selection and cross-validated AUC performance of three machine learning models based on the NCH dataset. In the left column of each panel, the importance of individual features was ranked, showing the top 20 features for each classifier. In the right column of each panel, the mean ROC curve (dark blue) was shown based on 10-fold cross-validation. Individual folds are multicolored, and shaded area denotes SD. **(A)** LASSO LR. **(B)** RF. **(C)** SVM classifier.

In feature selection, the LASSO LR features were chosen from nonzero coefficients determined by the model. Top features from LR include the mean spindle amplitude, spindle density, aperiodic intercept, and relative gamma power (Figure 1A). Feature ranking for RF was computed as the mean and standard deviation of accumulation of the impurity decrease within each tree. Top features from RF included SSO coupling strength, spindle density, aperiodic signal slope and intercept, relative gamma power, and REM sleep percent (Figure 1B). Similarly, top features from SVM included spindle amplitude, density, relative sigma power, and aperiodic intercept (Figure 1C). These three different methods yielded distinct top discriminating features due to differences in underlying nonlinearity and cost function.

We computed four sets of features: (i) hypnogram features of sleep staging and arousals; (ii) relative spectral power features; (iii) sleep spindle characteristics; and (iv) aperiodic signal characteristics (Table 2). For each feature set, relative contribution to performance of model classification was assessed. All categories decreased AUC indicating broad contribution of features. Exuding Spectral power had least impact on LR AUC performance at 0.62 vs. leave out aperiodic signal (0.53), macroarchitecture features (0.56), and spindle feature (0.55).

**Table 2:** Descriptive statistics of features included in the three machine learning models.

Feature group	Features	Control group mean (SD)	ASD group mean (SD)	*t*-test (value of p)
Polysomnogram/Macroarchitecture	Duration of Sleep Stage 1(h)	0.25 (0.22)	0.22 (0.21)	*t* = 0.68, *p* = 0.3
	Duration of Sleep Stage 2 (h)	2.8 (1.2)	1.7 (1.5)	*t* = 2.01, *p* = 0.04
	Duration of Sleep Stage 3 (h)	1.7 (0.75)	1.5 (1.1)	*t* = 1.4, *p* = 0.15
	Duration of REM (h)	1.1 (0.58)	0.8 (0.63)	*t* = 4.0. *p* = 0.00007
	Total Sleep Duration (h)	6.7 (2.6)	6.4 (2.3)	*t* = 0.56, *p* = 0.57
	Percentage Sleep Stage 1	40% (3.3%)	43% (4.9%)	*t* = 0.66, *p* = 0.5
	Percentage Sleep Stage 2	48% (22%)	51% (25%)	*t* = 0.8, *p* = 0.4
	Percentage Sleep Stage 3	26% (8.1%)	23% (4.1%)	*t* = 2.1, *p* = 0.04
	Percentage REM	18% (8.2%)	12% (7.6%)	*t* = 5.1, *p* < 0.0000003
	Number of REM Cycle	3.8 (2.5)	3.0 (2.4)	*t* = 2.4, *p* = 0.01
	EEG Arousal Count	35.1 (18)	31.8 (14)	*t* = 0.97, *p* = 0.3
Spectral features	Relative delta power	0.73 (0.08)	0.67 (0.14)	*t* = 3.4, *p* < 0.000005
	Relative theta power	0.14 (0.03)	0.14 (0.03)	*t* = 0.80. *p* = 0.40
	Relative alpha power	0.04 (0.02)	0.05 (0.02)	*t* = 1.17, *p* = 0.24
	Relative beta power	0.04 (0.03)	0.06 (0.04)	*t* = 4.1, *p* < 0.000005
	Relative sigma power	0.03 (0.02)	0.04 (0.04)	*t* = 4.6, *p* < 0.000005
	Relative gamma power	0.01 (0.01)	0.02 (0.04)	*t* = 4.5, *p* < 0.000005
Spindle/slow wave features	Mean spindle duration (s)	0.86 (0.33)	0.87 (0.42)	*t* = 0.22, *p* = 0.82
	Mean spindle frequency (Hz)	12.7 (0.3)	12.7 (0.3)	*t* = 0.96, *p* = 0.33
	Mean spindle amplitude (uV)	1,130 (851)	1,034 (732)	*t* = 0.91, *p* = 0.36
	Spindle density (# per min)	1.3 (0.13)	1.0 (0.02)	*t* = 2.3, *p* = 0.02
	Phase of SSO coupling	0.04 (0.07)	0.04 (0.07)	*t* = 0.42, *p* = 0.66
	Strength of SSO coupling	0.18 (0.01)	0.19 (0.01)	*t* = 1.7, *p* = 0.08
Aperiodic features	Aperiodic signal spectral slope	−2.2 (0.26)	−2.0 (0.34)	*t* = 2.86, *p* = 0.004
	Aperiodic signal spectral intercept	6.3 (0.78)	6.0 (0.88)	*t* = 2.3, *p* = 0.05

Spectral, spindle/slow wave features, and aperiodic features were computed from the average of all available EEG channels (F3-M2, F4-M1, C3-M2, C4-M1, O1-M2, O2-M1, and CZ-O1).

Each model had decreased AUC scores as compared to the AUC derived from the “full model.” Specifically, LR trained without spectral features resulted in an AUC of 0.62. LR trained without aperiodic features yielded an AUC of 0.53, while the AUC statistics without microarchitecture and spindle features were 0.56 and 0.52, respectively (Supplementary eTable S1).

### Model performance

Based on the full feature set, we trained three machine learning models separately and compared their AUC performances. In the NCH study, the 10-fold cross-validated AUC statistic varied between 0.80 and 0.95 (Figure 1C). The LR and SVM classifiers performed comparably across multiple metrics, with respective AUC 0.80 and 0.83, while the RF achieved a higher AUC of 0.95 (Table 3). Using an external dataset controls, the model achieved similar performance. We also tested the best model trained from the NCH study on the CHAT controls, and reported a true negative(TN) rate of 85% (67/79) and false positive (FP) rate of 15% (12/79). In the CHAT study, LR and SVM achieved an AUC between 0.83 and 0.87. Furthermore, none of the machine learning models were able to distinguish CHAT controls and NCH controls (Supplementary eTable S2). We also validated used the same methods of extracted features and machine learning models to examine a smaller younger autism patient population and age-matched controls (Supplementary eTable S2). LR showed reduced AUC performance [IQR: (0.65, 0.78)], and RF was comparable [IQR: (0.73, 0.99)].

**Table 3:** Performance [median (IQR)] comparison between three machine learning models based on 10-fold cross-validation.

Model	AUC	Sensitivity	Specificity	Accuracy	F1
NCH autism (*n* = 149) vs. NCH controls (*n* = 197)					
SVM	0.80 [0.78,0.85]	0.72 [0.60, 0.88]	0.69 [0.60, 0.80]	0.83 [0.78,0.86]	0.2 [0, 0.3]
RF	0.95 [0.93, 0.98]	0.74 [0.63, 0.90]	0.83 [0.75, 1.00]	0.94 [0.92,0.97]	0.76 [0.67, 0.89]
LR	0.83 [0.79,0.87]	0.93 [0.94,1.0]	0.87 [0.84,0.92]	0.82 [0.77,0.83]	0.52 [0.31,0.60]
NCH autism (*n* = 149) vs. CHAT controls (*n* = 79)					
SVM	0.87 [0.75,1.00]	0.97 [0.94,1.0]	0.83 [0.72,0.91]	0.83 [0.75,0.91]	0.88 [0.84, 0.94]
RF	0.85 [0.75,1.00]	0.96 [0.93,1.0]	0.86 [0.76,0.98]	0.89 [0.80,0.98]	0.9 [0.84, 0.96]
LR	0.83 [0.76,0.92]	0.93 [0.94,1.0]	0.87 [0.84,0.92]	0.84 [0.81,0.86]	0.89 [0.88, 0.93]

### Feature *post-hoc* analyzes

**Figure 2:**
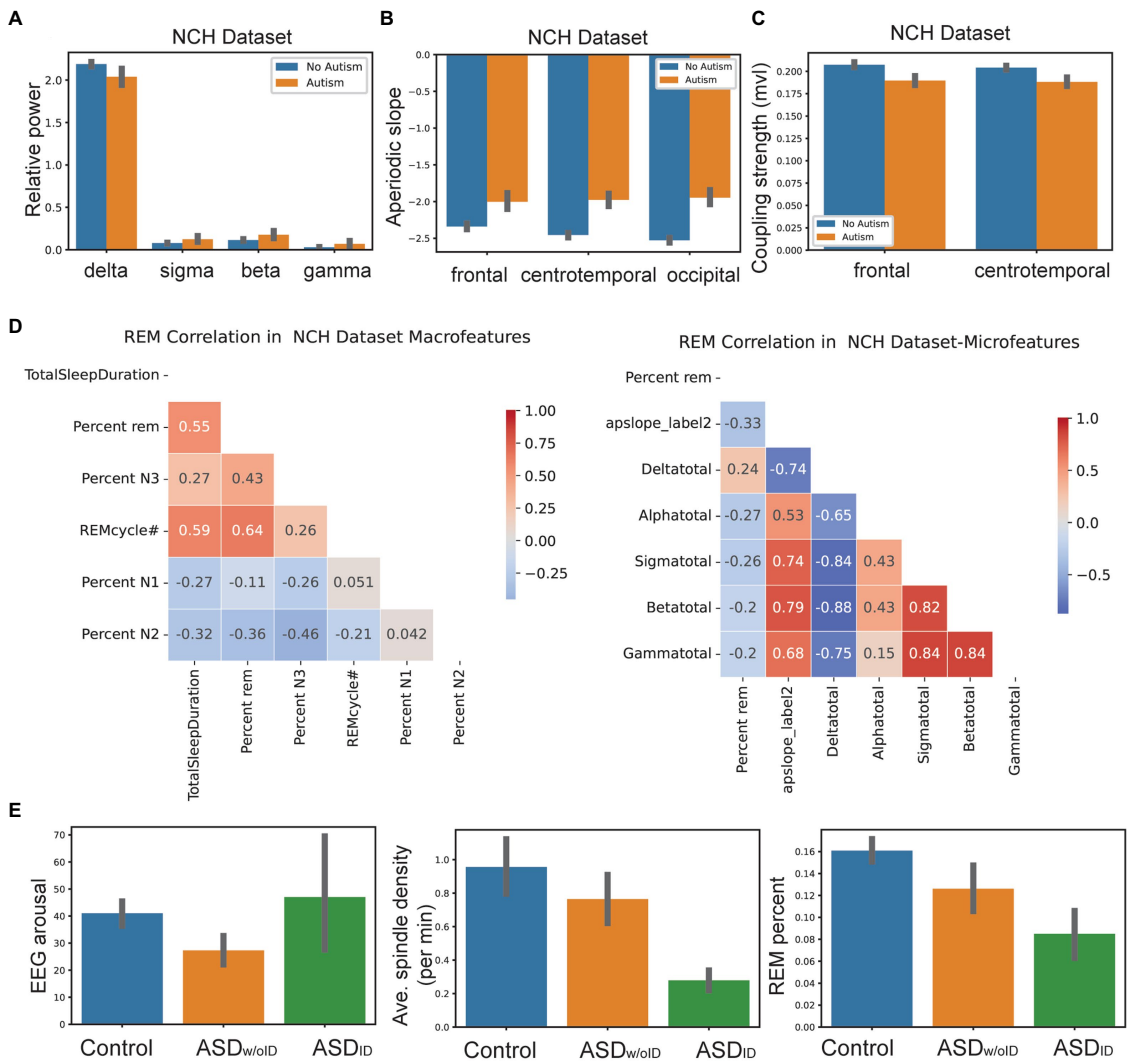
Machine learning analyses revealed future relationship within features and between features and clinical variables. **(A)** Relative spectral power in delta, sigma, beta and gamma frequency bands between NCH autism patients (*n*= 149) and controls (*n*= 197). Error bar denotes SEM. **(B)** Aperiodic slope in frontal, centro temporal and occipital leads between NCH autism patients and controls. Error bar denotes SEM. **(C)** SSO coupling strength in frontal and centro temporal leads between NCH autism patients and controls. Error bar denotes SEM. **(D)** Correlation between REM percentage with sleep macro and micro-architecture features in NCH patients. **(E)** Comparison of EEG arousal count, average spindle density and REM percentage features between controls, ASD_ID_ and ASD_w/oID_.

We identified most discriminative features from classification analysis. We found that first, many spectral features differed between autism and control groups. In the NCH dataset, the relative gamma, beta, sigma, and delta power was significantly different between autism and controls. NCH controls had increased relative delta and decreased relative gamma, beta, and sigma power (Figure 2A). However, the relative theta power (*t*-test: *t*-value = 0.8, *p* = 0.4), and alpha (*t*-value = 1.2, *p* = 0.2), did not show significant differences between NCH autism and controls.

Second, the aperiodic slope and aperiodic intercept were two highly ranked non-periodic features. Across EEG recording regions, NCH controls exhibited steeper (i.e., more negative) aperiodic slope compared to NCH autism patients. For instance, frontal region’s slope was -2.3 ± 0.17 (mean ± SD) in controls *vs* -2.0 ± 0.26 in NCH patients (t-test: *t*-value = 6.8, *p* = 8 × 10–7); temporal region’s slope was -2.5 ± 0.15 in controls *vs* -2.0 ± 0.31 in NCH patients (*t*-value = 8.6, *p* = 7 × 10–7); occipital region’s slope was -2.4 ± 0.28 in controls *vs* -1.9 ± 0.3 in NCH patients (*t*-value = 10.2, *p* = 3 × 10–7) (Figure 2B).

Third, in NCH controls, SSO coupling was stronger in frontal (mean phase-locking value 0.21 vs. 0.19 in patients, *t*-value = −6.1, *p* = 6 × 10–8) and central/temporal channels (mean 0.2 vs. 0.18 in patients, *t*-value = −6.3, *p* = 2 × 10–8) using single averaged channel comparisons (Figure 2C).

Fourth, decreased REM percent continued to be consistent feature in all autism patients in children and adults. REM percent was positively correlated with macroarchitecture features such as the total sleep duration and REM cycle #. There was a negative correlation between REM percent and NREM stages 1/2 in NCH patients. With respect to microarchitecture, REM percent had a weak negative correlation with relative spectral power in alpha, beta, sigma bands, and a positive correlation with relative delta power (Figure 2D).

Finally, CHAT controls showed similar sleep feature patterns as NCH controls. In the younger group (NCH infant/toddler vs. autism infant/toddler), there were similar patterns in all features except relative spectral power. In the young NCH dataset, there were differences in gamma, beta, and sigma power between autism and controls. However, unlike older ASD patients, young ASD patients showed a decrease in relative gamma, beta, and sigma power compared to young controls (Supplementary eFigure S2).

### Link to clinical variables

We further examined a subset of ASD population from the NCH dataset and divided them into two groups: with and without Intellectual Disability (ID): ASDID and ASDw/oID. We found that ASDID showed increased EEG arousal count with decreased spindle density and REM percent (Figure 2E). SSO coupling was increased in both ASDID and ASDw/oID. Comparisons of more features between ASDID and ASDw/oID are shown in Supplementary eTable S3. Finally, the prediction score was different between ASDID and ASDw/oID among NCH autism patients (*t*-value = 6.7, *p* = 0.0002; RF classifier).

In NCH controls, the peak spindle power occurred close relative to the SO trough (8 ms latency), whereas the latency was greater in ASDw/oID (20 ms) and ASDID (40 ms). Furthermore, spindle density was higher in controls compared to ASD children; EEG arousal was higher in ASDID than in ASDw/oID; REM sleep percent was highest in controls and lowest in ASDID (Figure 2).

## Discussion

Recent years have witnessed a rapid progress in biomarker discovery for ASD using various genetic and physiological markers (24–26). We combined feature engineering and machine learning tools to identify biomarkers for the ASD children population and validated the approach in two public datasets. The matched controls were a carefully selected group and did not likely contain neurodevelopmental diagnosis with the exception of attention-deficit/hyperactivity disorder (ADHD). Our results on independent validation demonstrated that machine learning achieved good generalizability and identified important sleep microarchitecture biomarkers.

Among sleep microarchitecture features, sleep spindles are consistently linked to cognitive abilities and memory consolidation, integrity of thalamocortical circuits, and sleep spindle abnormalities have been implicated in individuals with ASD, intellectual disability, and other neurodevelopmental disorders (27–29). Furthermore, sleep spindle density is linked to pace-making activity of the thalamic reticular nucleus and has been found to be stable across childhood; other spindle attributes show developmental trajectories throughout maturation (30). Our results showed decreased sleep spindle density and decreased SSO coupling in ASD children, consistent with the findings in the literature. We also found decreased aperiodic signal slope and increased aperiodic intercept during overnight sleep in ASD children. The spectral slope is thought to reflect the excitation/inhibition (E/I) balance at the synaptic level with more negative slopes reflecting enhanced inhibition, whereas the intercept is related to overall population spiking activity (31). A decrease in excitation during sleep in typical brain development has been associated with inhibitory regulation of cortical excitatory neurons *via* parvalbumin-positive (PV+) interneurons. The higher E/I ratio as evidenced by aperiodic slope flattening seen in ASD patients may confer a less stable sleep state, leading to decreased sleep stability and lower likelihood of entering REM sleep from NREM stages. NREM and REM total durations were negatively correlated in our study. It has been thought that NREM and REM are regulated independently of each other but are both under homeostatic control. Negative correlation between the two could be related to chronic sleep fragmentation in kids with autism, leading to NREM being favored at the expense of REM or disruption in the typical homeostatic REM sleep pressure which leads to rebound of REM sleep.

Among spectral features, there were specific band power differences between younger and older ASD patients. Younger patients (1–3 years) exhibited lower gamma power relative to controls, whereas older ASD children had increased gamma power relative to their age-matched controls. Since increased gamma is associated with deficient PV inhibition, it may suggest that early and persistent deficiencies of PV inhibition could explain abundance of gamma power in older ASD children (32, 33). To date, there is limited information regarding the ontology of gamma power in sleep in children. Gamma oscillations have been shown to be expressed throughout sleep in the human brain35. In ASD patients, gamma power increase during the resting state had been reported, particularly among patients with fragile X syndrome (34). Gamma rhythms have been attributed to synchronous activity of cortical PV interneurons. An increase in gamma power during sleep may be important for sensitive period closure in younger children (35). Early postnatal disruption of interneurons has been experimentally shown to cause increased cortical network synchrony (36). Therefore, increased gamma power may be associated with a reduced input to interneurons due to disrupted early circuit development, which, in turn, may shift E/I balance toward excitation and lead to disruptions in the shaping of cortical networks.

Overall, sleep microarchitecture features could serve as biomarkers of the deviant cortical maturation in autism. Spindle and aperiodic abnormalities signal thalamocortical dysfunction. Sleep microarchitecture disturbances may interfere with restoration of optimal E/I of neuronal homeostasis contributing to neurodevelopmental impairments (37). An improved understanding of neurophysiology of sleep brain networks may help advance the treatment of sleep disorders and underlying ASD pathophysiology.

### Limitations of the study

This study has some limitations. Diagnosis of autism and exclusion criteria were all based on ICD-10 coding and did not include gold standard autism assessments. There was also limited clinical and demographic information in the NCH database. The overall sample size was still relatively small, and the external validation dataset only included a healthy control group. Additionally, type-I errors may exist in our machine learning analyzes. Our independent validation with CHAT controls used the same ASD group as the NCH validation and therefore did not allow us to confirm autism group results. Finally, there was a degree of selection bias since all children were referred for sleep study in most cases due to suspicion of sleep disordered breathing at the first place.

## Conclusion

Machine learning can identify EEG sleep biomarkers that distinguish children with clinically diagnosed autism from typically developing controls. Feature ranking revealed important discriminative sleep microarchitecture features. Sleep disturbances are among the most pronounced challenges faced by ASD children and their families; our machine learning models may provide a pragmatic next step for clinical intervention.

## Data availability statement

The original contributions presented in the study are included in the article/Supplementary material, further inquiries can be directed to the corresponding author.

## Ethics statement

The studies involving human participants were reviewed and approved by data collected as part of NSRR databank. Written informed consent to participate in this study was provided by the participants’ legal guardian/next of kin.

## Author contributions

CM had full access to all of the data in the study, took responsibility for the integrity of the data and the accuracy of the data analysis, drafting of the manuscript, and statistical analysis. CM and ZC contributed to the concept and design, acquisition, analysis or interpretation of data. ZC took responsibility for critical revision of the manuscript for important intellectual content, funding, and supervision. All authors contributed to the article and approved the submitted version.

## Funding

This study was partially funded by grants from the US National Institute of Mental Health (MH118928), National Institute of Neurological Disorders and Strokes (NS123928 and NS123928S1), National Institute of Drug Abuse (DA056394), and National Science Foundation (CBET-1835000).

## Conflict of interest

ZC reports grants from the National Institutes of Health (NIH) and National Science Foundation (NSF) during the conduct of the study. ZC is also a founder and CEO of NeuroThX, LLC. ZC also received cloud computing resources supported by the Oracle for Research Award.

The remaining author declares that the research was conducted in the absence of any commercial or financial relationships that could be construed as a potential conflict of interest.

## Publisher’s note

All claims expressed in this article are solely those of the authors and do not necessarily represent those of their affiliated organizations, or those of the publisher, the editors and the reviewers. Any product that may be evaluated in this article, or claim that may be made by its manufacturer, is not guaranteed or endorsed by the publisher.

## Footnotes

^1^ https://mne.tools/stable/index.html

^2^ https://pypi.org/project/yasa/

^3^ https://pingouinInreviewstats.org/index.html

^4^ https://fooof-tools.github.io/fooof/index.html
