# Supplementary material for: Identification of atypical sleep microarchitecture biomarkers in children with autism spectrum disorder
Source: Front Psychiatry. 2023 Apr 17;14:1115374. doi: 10.3389/fpsyt.2023.1115374 (PMC10150704; doi:10.3389/fpsyt.2023.1115374)

Supporting Information

eTable 1: Performance of LASSO-LR model based on the leaving-one-feature-set-out procedure.

Leave-one-feature-set-out	AUC	Sensitivity	Specificity	Accuracy
Spectral Power	0.62	0.31	0.56	0.81
Aperiodic Signal	0.53	0.12	0.99	0.81
Macroarchitecture	0.56	0.19	0.94	0.80
Sleep Spindle	0.55	0.19	0.91	0.77

eTable 2: Performance summary of three machine learning models (median [IQR]) in two additional validation studies. In the first validation, the LR classifier was trained to discriminate NCH controls from CHAT controls. In the second validation, the LR classifier was trained to discriminate a smaller independent sample from the NCH dataset.

Model	AUC	Sensitivity	Specificity	Accuracy
NCH Controls (n=197) vs. CHAT Controls (n=79)				
SVM	0.45 [0.2,0.7]	0 [0,0.3]	0 [0,0]	0.7 [0.5, 0.8]
RF	0.61 [0.4,0.7]	0 [0,0]	0.03 [0,0]	0.5 [0.4,0.7]
LR	0.47 [0.00,0.75]	0.25 [0,0.38]	0.25 [0.00,0.37]	0.60 [0.42,0.75]
Independent NCH dataset: Autism (n= 38) vs. Controls (n=75)				
SVM	0.70 [0.63, 0.98]	0.5 [0.2, 0.6]	0.7 [0.5, 0.8]	0.78 [0.73, 0.85]
RF	0.82 [0.73,0.99]	0.4 [0.1, 0.6]	0.6 [0, 1.0]	0.87 [0.8, 0.93]
LR	0.74 [0.65,0.78]	0.4 [0.2,0.7]	0.6 [0.66,0.97]	0.87 [0.8,0.93]

eTable 3: Comparison of sleep EEG features among the autistic children (NCH dataset) with vs. without intellectual disability (ID).

Sleep EEG Features	Autistic children with ID (n=20), mean (SD)	Autistic children without ID (n=129), mean (SD)	t-test (p-value)

Mean spindle amplitude (uV; frontal)	1283 (115)	686 (166)	t-value=4.1 (p=0.007)
EEG arousal count	44.5 (27)	27.2 (20)	t-value=2.89 (p=0.02)
Spindle density (# per min, central)	0.3 (0.1)	0.7 (0.3)	t-value=3.7 (p=0.002)
Percent REM sleep	8(5)	13(6)	t-value=2.8 (p=0.006)

eFigure 1. Schematic overview of the study design.

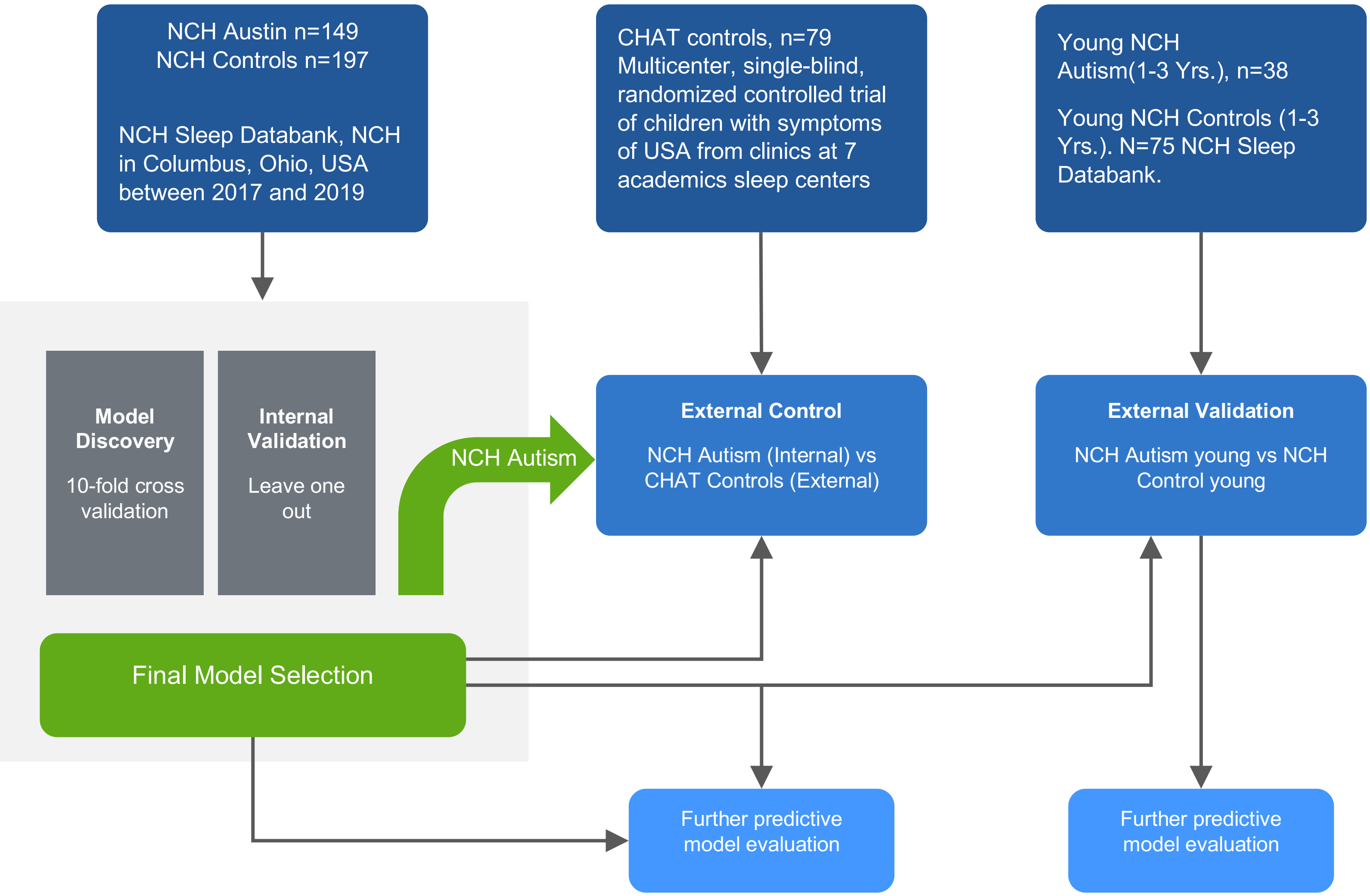

eFigure 2. Additional comparison of relative spectrum power features.

(A) Relative spectral power in delta, sigma, beta and gamma frequency bands between NCH autism patients (n=149) and CHAT controls (n=79). Error bar denotes SEM.

(B) Relative spectral power in delta, sigma, beta and gamma frequency bands between young NCH autism patients (n=38) and controls (n=75). Error bar denotes SEM.

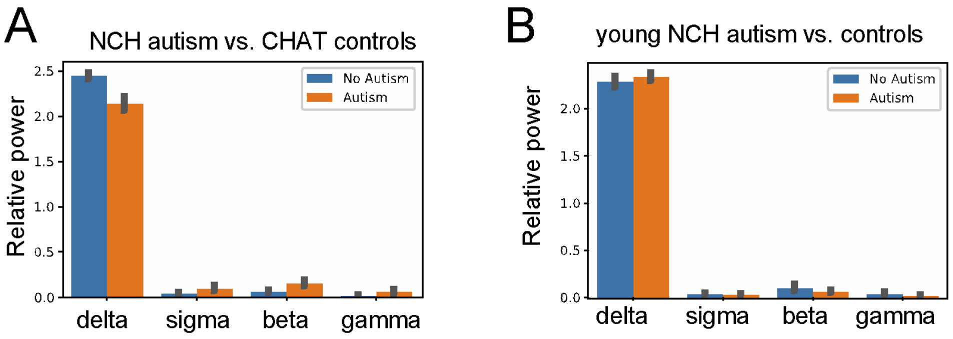

eFigure3 Violin plots of data distributions

Distribution plots of pca based outlier detection method. Outlier scores are calculated as the sum of the weighted euclidean distance between the data point to the low-dimensional hyperplane. The outlier threshold indicates data points falling outside of Interquartile range of 1-3.

(A) Outlier distribution of NCH data features. Outlier threshold=1.25
(B) Outlier distribution of CHAT data features. Outlier threshold=1.5
(C) Outlier distribution of NCHyoung data features. Outlier threshold=0.8

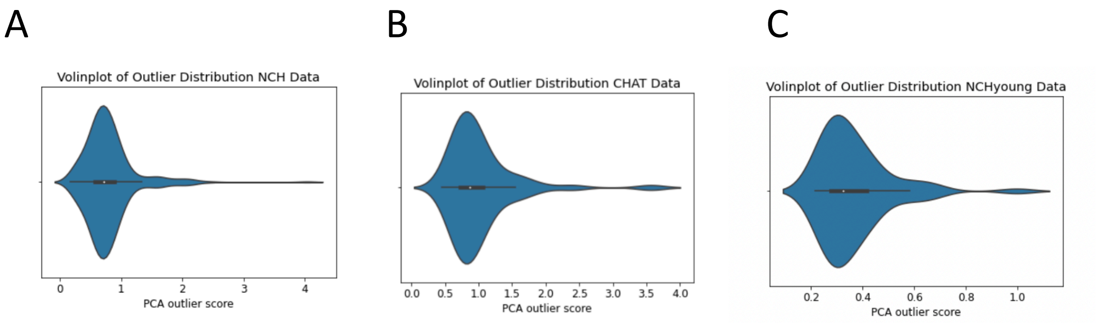

Supplement: Supplementary file 1 [file Data_Sheet_1.PDF]
